# Comprehensive Volatile Metabolite Profiling and Bioactive Marker Discovery of Saposhnikoviae Radix via HS-GC-MS/MS Integrated with Chemometrics and Network Pharmacology

**DOI:** 10.3390/metabo16090689

**Published:** 2026-09-18

**Authors:** Fangliang He, Xianrui Wang, Jiating Zhang, Lizhi Wan, Haonan Wu, Xianlong Cheng, Jia Chen, Xiangri Li, Wenguang Jing, Feng Wei

**Affiliations:** 1 Traditional Chinese Medicine Processing Technology Inheritance Base of National Administration of Traditional Chinese Medicine, Beijing University of Chinese Medicine, Beijing 102488, China; hefangliang0602@126.com; 2 Beijing Key Laboratory for Quality Evaluation of Chinese Materia Medica, Beijing University of Chinese Medicine, Beijing 102488, China; 3 Institute for Control of Traditional Chinese Medicine and Ethnic Medicine, National Institutes for Food and Drug Control, Beijing 102629, China; wangxianrui@nifdc.org.cn (X.W.); 18341441178@163.com (J.Z.); wanlizhi@nifdc.org.cn (L.W.); 19861403424@163.com (H.W.); cxl@nifdc.org.cn (X.C.); chenjia@nifdc.org.cn (J.C.)

**Keywords:** Saposhnikoviae radix, HS-GC-MS/MS, volatile metabolomics, chemometrics, network pharmacology, molecular docking, biomarker discovery, anti-inflammatory

## Abstract

**Background: **Saposhnikoviae radix (SR), a valuable Chinese medicinal plant with high medicinal and edible value, suffers from inconsistent quality caused by growth patterns, cultivation duration, and geographical origin. **Methods:** In this study, comprehensive volatile metabolite profiling using HS-GC-MS/MS combined with multivariate chemometrics was performed to characterize these quality-related differences. **Results:** In total, 7, 13, and 11 differential volatile compounds were identified for wild versus cultivated SR, cultivation duration, and geographical origin, respectively. By integrating the three comparisons, 11 recurrent differential compounds were selected for network pharmacology and molecular docking analyses. trans-3-Nonen-2-one, benzaldehyde, and Octanoic acid were predicted to interact with key targets, including TNF, IL1B, ALB, EGFR, and CASP3, which were mainly enriched in inflammation- and pain-related pathways, including the PI3K-Akt signaling pathway, apoptosis, and the HIF-1 signaling pathway. Molecular docking further supported favorable predicted interactions between the selected compounds and core targets. **Conclusions:** Overall, this study reveals volatile metabolite variation associated with major quality-related factors of SR and identifies candidate bioactive and quality-associated volatile compounds. The integration of volatile metabolite profiling, chemometrics, network pharmacology, and molecular docking provides a complementary strategy for the comprehensive quality evaluation of SR and offers a reliable reference for quality control research on other medicinal Apiaceae species.

## 1. Introduction

Saposhnikoviae radix (SR), the dried root of *Saposhnikovia divaricata* (Turcz.) Schischk., was first listed in the Shennong Bencao Jing as a top-grade, valuable Chinese medicinal crude drug with high medicinal and edible value. Researchers have isolated many practical components from SR, such as chromones, coumarins, acid esters, polysaccharides, volatile oils, and other compounds. Modern pharmacological studies have shown that the volatile oil has anti-inflammatory and analgesic properties and is one of the main active ingredients in SR [1,2,3]. However, many factors, such as whether the plant is a wild or cultivated product, different cultivation durations, and its geographical origins, make a big difference in its active ingredients [4,5]. Therefore, there is an urgent need to identify potency-related compounds as quality control markers to evaluate the quality of SR.

The existing quality control methods cannot entirely control the quality of SR and reflect its embodied overall therapeutic efficacy. The current quality control of SR is primarily based on pharmacopoeial marker-based quality control. To obtain more comprehensive chemical information, various analytical techniques and evaluation strategies have been applied to investigate the chemical characteristics and quality variation in SR. For example, Chen et al. established an HPLC fingerprint combined with chemometric analysis to evaluate SR samples from different geographical origins [6]. Yue et al. further developed an HPLC fingerprint for the lipophilic components of SR and compared samples collected from different regions [7]. In addition, Fuchino et al. employed UHPLC/MS for the quantitative analysis and comparison of SR constituents among cultivated, wild, and commercial products, with particular attention to differences associated with growth pattern and cultivation duration [4]. Xu et al. applied GC-MS and UPLC-MS/MS to compare the chemical constituents of wild, wild-simulated, and cultivated SR, thereby investigating chemical variation associated with different growth patterns [8]. Moreover, Chen et al. developed an integrated UHPLC-QTOF-MS strategy for the comprehensive characterization of chromones and coumarins in SR samples from different geographical regions [9]. Collectively, these studies demonstrate that HPLC, UPLC, GC-MS, and LC-MS have been increasingly applied to the chemical characterization and quality evaluation of SR. However, previous studies have generally focused on chromones, coumarins, and other nonvolatile constituents and generally investigate individual quality-related factors. Studies specifically investigating volatile chemical variation in SR remain comparatively limited, particularly systematic investigations of volatile chemical variation associated with growth patterns, cultivation duration, and geographical origin. Therefore, further characterization of the volatile profiles of SR may provide complementary chemical information for a more comprehensive understanding of its quality variation.

In recent years, the combination of multi-technology has become a trend [10]. As a technique for detecting volatile compounds, headspace gas chromatography–mass spectrometry (HS-GC-MS/MS) is a simple and high-speed method that eliminates the need for sample pre-treatment [11]. The network pharmacology of traditional Chinese medicine (TCM) is suitable for the characteristics of the multi-target and complex pharmacological action of TCM and has provided an excellent predictive platform for the mechanistic study of TCM [12,13,14]. Molecular docking technology uses virtual screening technology to find novel drugs’ action targets and study the binding mode between ligands and receptors [15,16]. The combination of network pharmacology and HS-GC-MS/MS can both control the quality of TCM as a whole and reveal the material basis and pathways for TCM to exert its effects.

In this study, we hypothesized that the volatile chemical profile of SR is influenced by growth pattern, cultivation duration, and geographical origin, and that volatile compounds identified as differential components may represent candidate quality-associated compounds with potential relevance to the anti-inflammatory and analgesic activities of SR. Accordingly, the specific objectives of this study are clearly stated, including the characterization of SR volatile profiles using HS-GC-MS/MS, the evaluation of the effects of the three quality-associated factors using multivariate statistical analysis, and the screening of candidate compounds followed by network pharmacology and molecular docking analyses to predict their potential anti-inflammatory and analgesic mechanisms. By integrating volatile chemical profiling with chemometric and bioinformatics approaches, this study aims to reveal volatile metabolite variation associated with major quality-related factors of SR, identify candidate bioactive and quality-associated volatile compounds, and provide a complementary strategy for the comprehensive quality evaluation of SR, as well as a reference for quality control research on other medicinal Apiaceae species.

## 2. Materials and Methods

### 2.1. Herbal Materials

A total of 140 SR specimens were collected from different geographical regions, including Inner Mongolia, Heilongjiang, and Hebei Province in China, as well as Mongolia and the Russian Federation. The collection comprised 120 cultivated and 20 wild samples. The cultivated materials covered several cultivation durations, including 1-, 2-, and 3-year-old seed-sown plants and seedling-transplanted plants with cultivation periods of 1 + 1, 2 + 1, 3 + 1, and 1 + 3 years. For each sampling site, multiple individual plants were collected from the same field plot, pooled randomly, and subsequently divided into five or ten portions. Detailed information on the sample sources, collection location, growth patterns, cultivation methods, cultivation duration and collection date of each sample is provided in Table S1.

All SR samples were identified by Prof. Feng Wei (Institute for National Institutes for Food and Drug Control, Beijing, China) according to the requirements of the 2020 edition of the Chinese Pharmacopoeia. Following collection, the samples were naturally dried under ambient conditions. The dried materials were ground into powder and passed through a 50-mesh sieve before HS-GC-MS/MS analysis. The prepared samples were analyzed immediately, while the remaining materials were stored in a cool, dry, and light-protected place.

### 2.2. Sample Preparation

For each analysis, 0.5 g of powdered SR sample was accurately weighed and transferred into a 10 mL headspace vial. A pooled quality control (QC) sample was prepared by combining equal aliquots of all individual samples and was used as the reference for data normalization. Before GC-MS/MS analysis, the vials were equilibrated under the following headspace conditions: thermostat temperature, 120 °C; sample flow path temperature, 150 °C; transfer line temperature, 140 °C; and vial equilibration time, 10 min.

### 2.3. HS-GC-MS/MS Analysis

Volatile constituents were analyzed using a Shimadzu GCMS-TQ8050 NX triple quadrupole gas chromatograph–mass spectrometer (GC-MS/MS) coupled with an HS-10 headspace injector (Shimadzu, Kyoto, Japan). The chromatographic conditions were adapted from previously published HS-GC-MS procedures for volatile compound analysis, with modifications made according to the characteristics of the SR samples and the configuration of the instrument used in this study [17,18,19,20].

Chromatographic separation was achieved on an HP-5MS fused silica capillary column (30 m × 0.25 mm, 0.25 µm) using helium as the carrier gas at a constant flow rate of 1 mL·min^−1^. The temperature was initially set to 50 °C and maintained for 2 min, increased to 120 °C at 10 °C·min^−1^, and then raised to 170 °C at 2.5 °C·min^−1^. The temperature was finally increased to 240 °C at 10 °C·min^−1^ and held for 3 min. The total run time was 45 min. The injector temperature was set at 250 °C. Samples were introduced in split mode at a split ratio of 10:1, with an injection volume of 1 µL.

Mass spectrometric detection was conducted using an electron bombardment ion source (EI) at an electron impact energy of 70 eV. The interface and ion source temperatures were set at 280 °C and 230 °C, respectively. Data were acquired in full-scan mode over an *m*/*z* range of 20–650.

### 2.4. Network Pharmacology Analysis

The effective ingredients were screened based on the analysis results from HS-GC-MS/MS. Candidate volatile compounds selected from the HS-GC-MS/MS-based chemometric analysis were subjected to target prediction. The TCMSP (https://www.tcmsp-e.com/tcmsp.php (accessed on 15 September 2026)), superPRED (https://prediction.charite.de/ (accessed on 15 September 2026)), HERB (http://herb.ac.cn/), SEA (https://sea.bkslab.org), CTD (https://ctdbase.org/) and Target NET (http://targetnet.scbdd.com/) databases were searched to obtain potential compound-associated targets [21,22]. The retrieved targets were subsequently standardized to human gene identifiers using the UniProt (http://www.Uniprot.org/) database, and duplicate entries were removed. Potential targets associated with analgesic and anti-inflammatory processes were collected from the Gene Cards (https://www.genecards.org/), DisGeNET (https://www.disgenet.org/), and CTD (https://ctdbase.org/) databases using the search terms “pain” and “inflammation”. The resulting disease-related target sets were combined, followed by the removal of duplicate entries.

The compound-associated and disease-associated target sets were intersected using Venny 2.1 (https://bioinfogp.cnb.csic.es/tools/venny/ (accessed on 15 September 2026)) to obtain candidate common targets. These targets were subsequently imported into the STRING (https://string-db.org/) database to construct the Protein–Protein Interaction (PPI) network, and hub targets were identified using the Analyze Network plug-in software. Gene Ontology (GO) and the Kyoto Encyclopedia of Genes and Genomes (KEGG) enrichment analyses were subsequently performed using the DAVID (https://davidbioinformatics.nih.gov/ (accessed on 15 September 2026)) platform with human genes as the reference species. The enriched GO terms and the top 20 KEGG pathways were visualized using the Bioinformatics platform (https://www.bioinformatics.com.cn/). Finally, Cytoscape 3.10.1 software was used to construct the compound–target–pathway network, and degree values were considered when identifying compounds, targets, and pathways.

### 2.5. Molecular Docking Verification

Molecular docking was performed to further investigate the predicted interactions between the candidate volatile compounds and selected hub targets. Protein structures were obtained from the PDB database, and the corresponding 3D structures of the ligands were downloaded from the PubChem database in SDF format. Open Babel was used to convert the ligand structures into PDBQT format. Before docking, the receptor and ligand were processed using AutoDockTools 1.5.6, including water removal, hydrogen addition, partial-charge assignment and rotatable-bond definition, and the default settings of the software were applied where applicable [23]. Semi-flexible docking was carried out using AutoDock 4.2. The docking grid box was defined as 120 × 120 × 120 points with a spacing of 0.375 Å. Among the generated docking conformations, the pose with the lowest energy was selected for subsequent structural visualization using PyMOL 2.3.0 to characterize hydrogen-bond interactions between compounds and target residues. The lowest-energy docking pose was selected and visualized.

### 2.6. Data Processing and Multivariate Statistical Analysis

The raw MS data were processed with Progenesis QI software (version 2.3), which was used for peak alignment and data normalization, followed by application of its default normalization procedure. The resulting normalized abundance matrix was exported for subsequent statistical analysis. Multivariate analyses were then conducted using SIMCA-P 14.1 (Umetrics, Umeå, Sweden).

## 3. Results

### 3.1. Volatile Compound Analysis by HS-GC-MS/MS

The data obtained from HS-GC-MS/MS were compared with the standard spectral library NIST17, and compounds with ≥90% match were screened out. After combining relevant literature reports [24,25,26,27] and online databases, a total of 62 volatile compounds were finally tentatively tentatively identified in SR samples (Table S2). Progenesis QI was used to process the raw dataset, followed by multivariate analysis in SIMCA-P 14.1.

#### 3.1.1. Comparison of Volatile Compounds Between Wild and Cultivated SR

Principal component analysis (PCA), partial least squares–discriminant analysis (PLS-DA), and orthogonal partial least squares–discriminant analysis (OPLS-DA) are classical dimensionality reduction models in data pre-processing methods that can convert a large number of variables into several synthetic variables [28,29,30]. The OPLS-DA model is a supervised discriminant analysis method, which can separate the information irrelevant to preset and classification from the original matrix to the greatest extent, thus weakening the intra-group differences and maximizing the inter-group differences [31,32]. With the OPLS-DA model, wild and cultivated SR were separated from each other compared to the PCA or PLS-DA model clearly.

**Figure 1:**
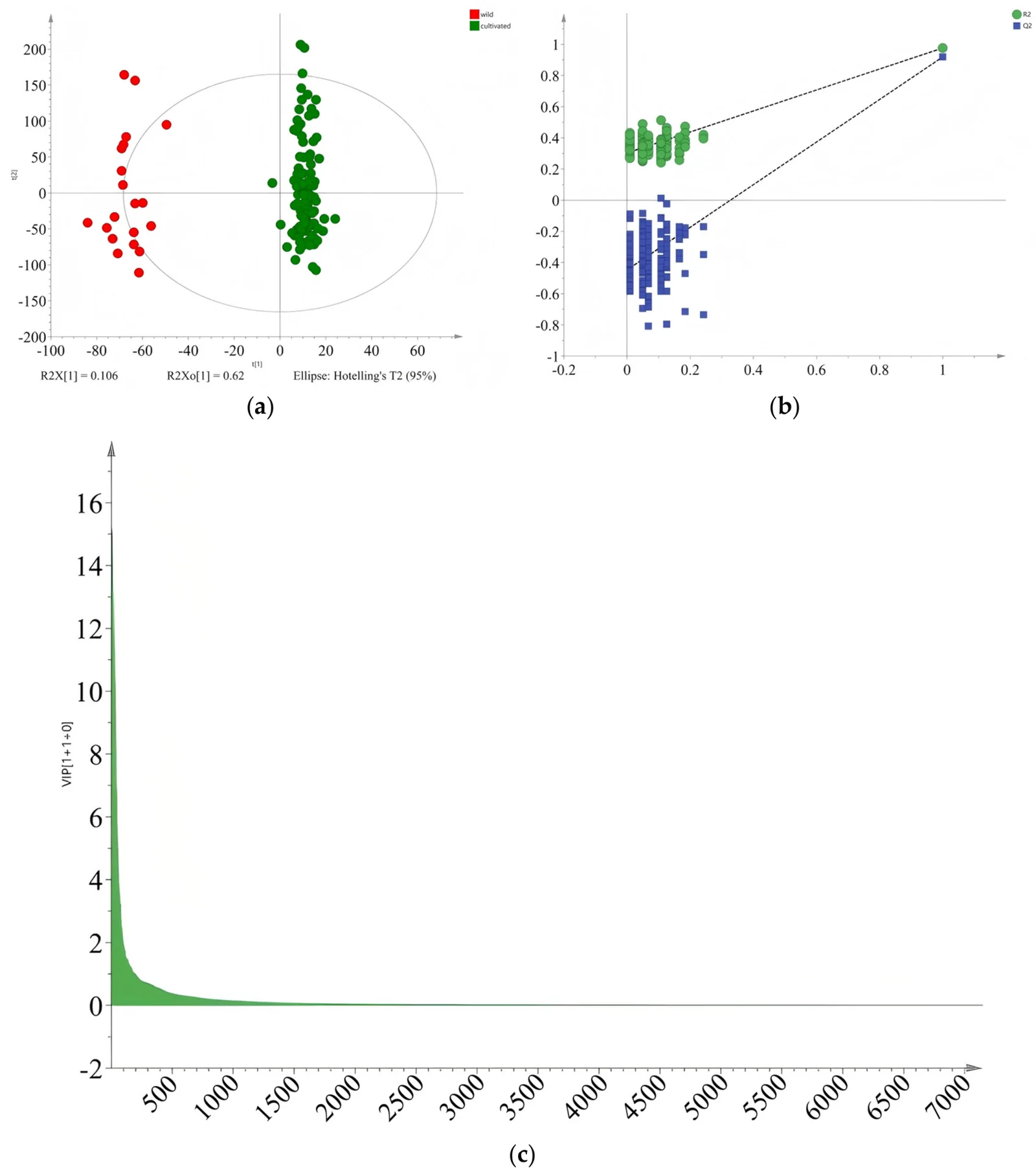
Results of principal compound analysis between wild and cultivated SR. (**a**) Scores plot for OPLS–DA model; (**b**) substitution test chart of OPLS–DA model (dashed lines represent the linear regression lines of R^2^Y and Q^2^); (**c**) VIP chart of OPLS–DA model.

The OPLS-DA model was applied to distinguish wild and cultivated SR, and the vital volatile compounds were identified according to VIP values. Figure 1a clearly showed that the wild and cultivated samples were separated from each other. The interpretation rate parameter R^2^Y of the OPLS-DA model was 0.828, and the predictive ability parameter Q^2^ was 0.775; both are greater than 0.5, proving the model is stable. This suggests that the OPLS-DA model had a reliable performance. At the same time, to avoid over-fitting in the OPLS-DA model, we employed the permutation test (*n* = 200) using SIMCA 14.1 to assess the model’s reliability. From Figure 1b, it can be observed that even after 200 rounds of cross-validation, the response line of model Q^2^ still intersected with the *x*-axis and had an intercept (−0.447) smaller than 0 with the *y*-axis, indicating that the model was not subject to over-fitting [33], signifying that the OPLS-DA model established in this paper was stable, reliable, and statistically significant.

In principle, the greater the variable importance in projection (VIP) value, the greater the contribution of the component to group classification; moreover, VIP > 1.0 is often considered as a commonly used criterion for screening differential components [34]. In order to make the results of differential component analysis more reliable and accurate, we make use of a nonparametric rank sum test (data not normally distributed) to verify whether there was a significant difference. In Figure 1c, seven volatile compounds are identified as vital compounds for distinguishing wild and cultivated SR, with VIP > 1.0 and a probability value (*p*) ≤ 0.05. They are β-Bisabolene, Octanoic acid, benzaldehyde, panaxynol, trans-3-Nonen-2-one, (E)-2-nonanal and 1-Heptano.

#### 3.1.2. Comparison of Volatile Compounds in SR of Different Cultivation Duration

**Figure 2:**
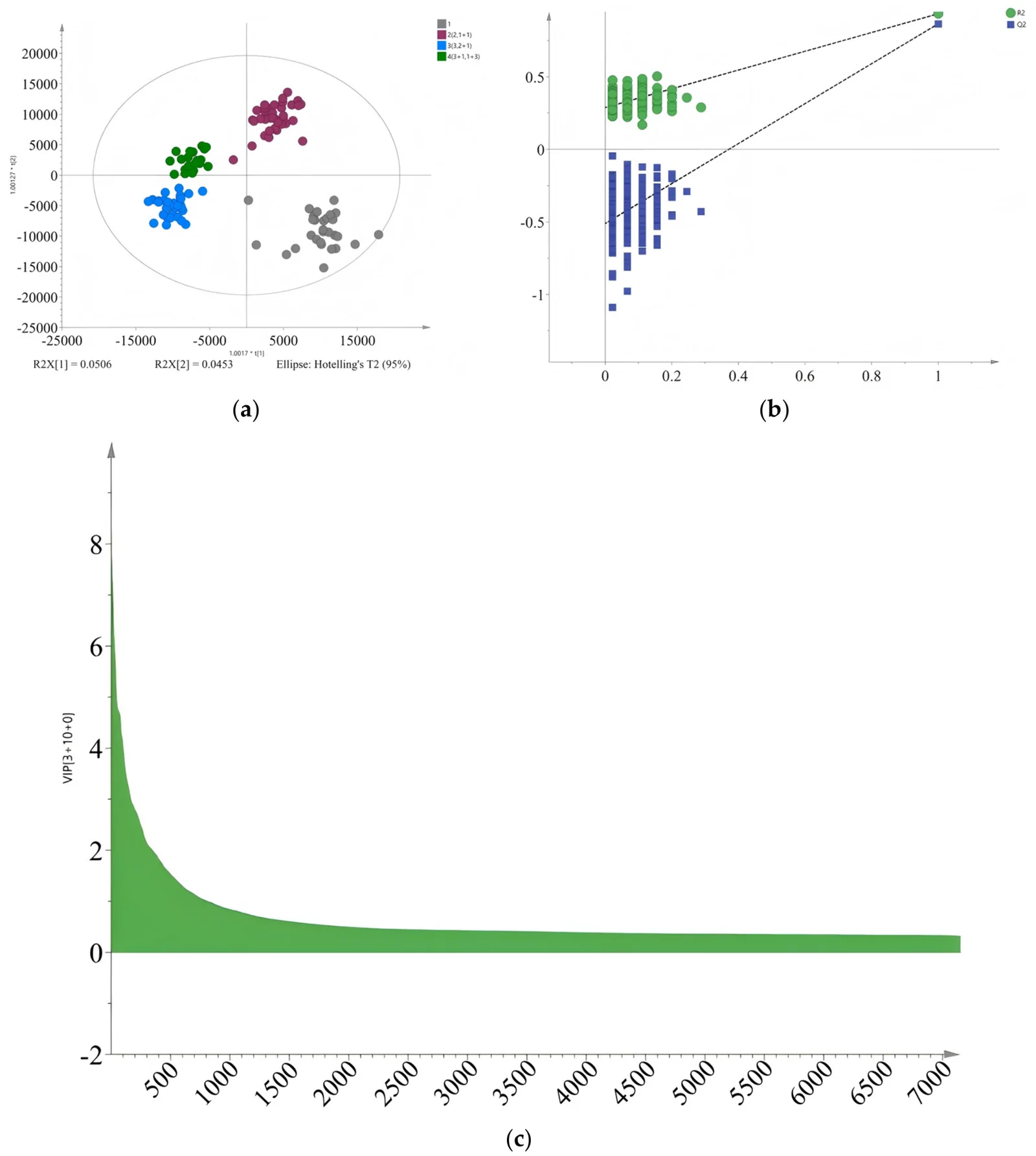
Results of principal compound analysis in SR of different cultivation duration. (**a**) Score plot for OPLS–DA model; (**b**) substitution test chart of OPLS–DA model (dashed lines represent the linear regression lines of R^2^Y and Q^2^); (**c**) VIP chart of OPLS–DA model.

The collected cultivar samples can be categorized by year as 1 (seed sowing for 1 year), 2 (seed sowing for 2 years and seed sowing 1 year, transplanting 1 year), 3 (seed sowing for 3 years and seed sowing 2 years, transplanting 1 year), and 4 (seed sowing 3 years, transplanting 1 year and seed sowing 1 year, transplanting 3 years) years old. We used the OPLS-DA model to identify critical volatile compounds that are responsible for the volatile compounds of the different cultivation durations (Figure 2). The cumulative explanatory power parameters R^2^X and R^2^Y of the model are 0.905 and 0.923, respectively, and the predictive power parameter Q^2^ is 0.843. All three parameters are greater than 0.5, proving the model is stable. Meanwhile, from Figure 2b, the current model was validated using the permutation test (*n* = 200), with R^2^ = (0, 0.288) and Q^2^ = (0, −0.511). Compared to the original values on the right side, the values on the left side of the fitted straight lines of R^2^ and Q^2^ generated by random permutation were smaller, and the fitted straight line intercept of Q^2^ was negative, signifying that the OPLS-DA model established in this paper was stable, reliable, and statistically significant.

With VIP > 1.0 and *p* ≤ 0.05, we preliminary identified 13 vital volatile compounds that may be potential difference components for distinguishing between SR of different cultivation durations. The VIP chart of the OPLS-DA model is shown in Figure 2c. These volatile compounds were β-Bisabolene, panaxynol, (E)-2-nonanal, Benzene,1-methyl-3-(1-methylethyl)-, 1-Heptano, trans-3-Nonen-2-one, benzaldehyde, (E)-2-Octenal, 2-octenal,2-butyl, cedrene, (E,E)-2,4-nonadienal, hexanal and decana.

#### 3.1.3. Comparison of Volatile Compounds in SR from Different Geographical Origins

**Figure 3:**
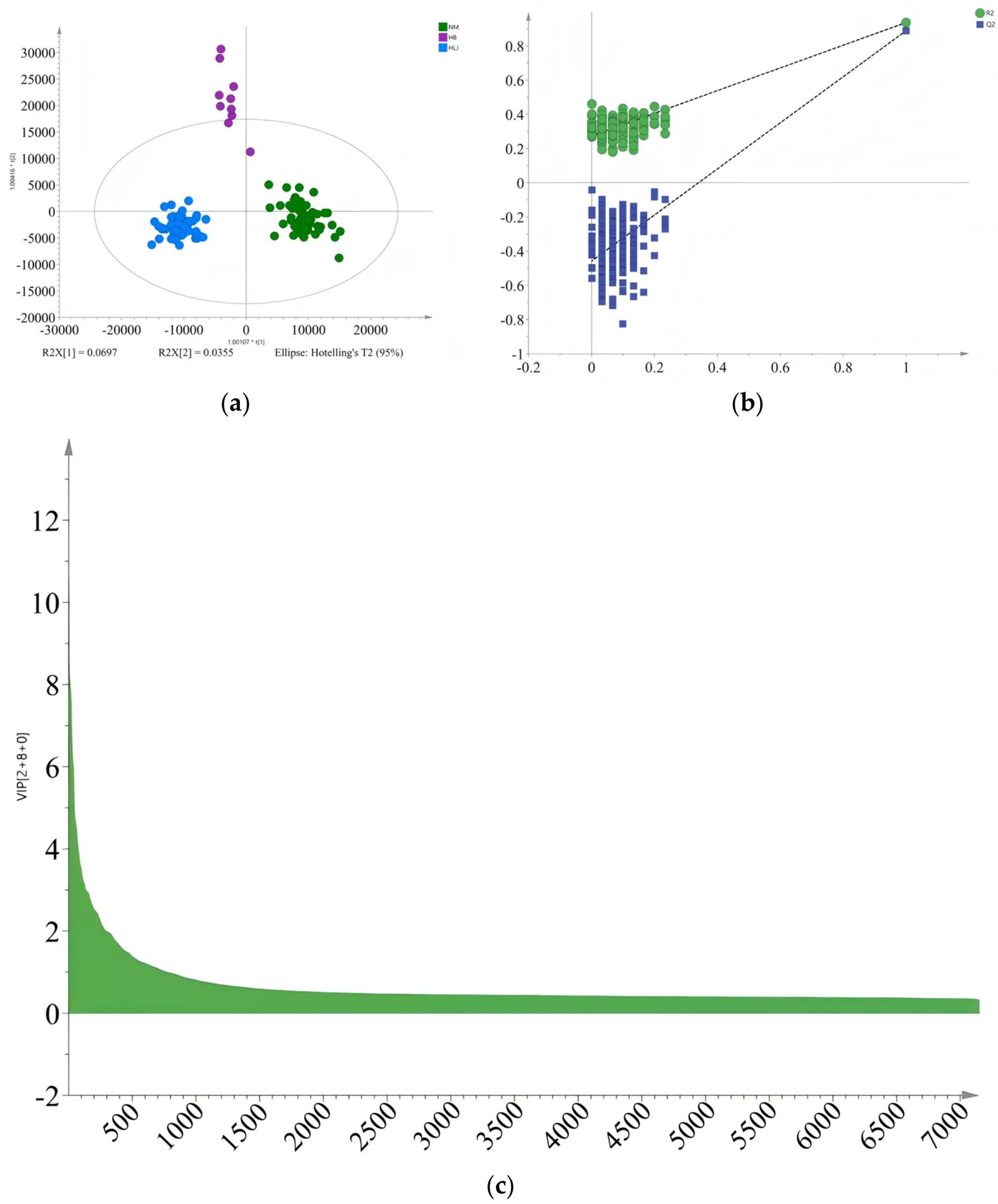
Results of principal compound analysis in SR from different geographical origins. (**a**) Score plot for OPLS–DA model; (**b**) substitution test chart of OPLS–DA model (dashed lines represent the linear regression lines of R^2^Y and Q^2^); (**c**) VIP chart of OPLS–DA model.

Cultivated SR species were collected from Inner Mongolia, Heilongjiang, and Hebei. In order to more intuitively visualize the differences between different geographical origins, OPLS-DA models were applied to distinguish between Inner Mongolia, Heilongjiang, and Hebei (Figure 3). The model’s cumulative explanatory power parameters R^2^X and R^2^Y are 0.847 and 0.914, respectively, and the predictive power parameter Q^2^ is 0.844. All parameters are greater than 0.5, signifying that the model is stable. The OPLS-DA analysis result is shown in Figure 3a. From Figure 3b, the current model was validated using the permutation test (*n* = 200), with R^2^ = (0, 0.273) and Q^2^ = (0, −0.458), and had an intercept smaller than 0 with the *y*-axis, indicating that the model was not subject to over-fitting, proving that the OPLS-DA model established in this paper was stable, reliable, and statistically significant.

The VIP chart of the OPLS-DA model is shown in Figure 3c; a total of 11 vital volatile compounds were preliminary identified as vital compounds for distinguishing between different geographical origins, with VIP > 1.0 and *p* ≤ 0.05. These were β-Bisabolene, Octanoic acid, 1-Heptano, benzaldehyde, cedrene, (E,E)-2,4-nonadienal, undecanal, octenal,2-butyl, Octanoic acid, methy ester and (E)-2-Octenal.

### 3.2. Network Pharmacology Results

#### 3.2.1. Target Prediction of Candidate Compounds and Diseases

To identify shared differential compounds associated with multiple comparisons, the differential compound lists obtained from the three comparisons were compared. Compounds that were repeatedly identified in at least two of the three comparisons were retained for subsequent network pharmacology analysis (Figure S1). A total of 11 candidate volatile compounds were finally selected: benzaldehyde, 1-Heptano, β-Bisabolene, trans-3-Nonen-2-one, (E)-2-nonanal, panaxynol, Octanoic acid, Benzene,1-methyl-3-(1-methylethyl)-, (E)-2-Octenal, (E,E)-2,4-nonadienal, and cedrene (Table S3).

To identify the potential targets of SR volatile compounds involved in anti-inflammatory and analgesic effects, we performed target prediction for the 11 candidate compounds using the TCMSP, HERB, CTD and target NET databases. For each database, the search was restricted to *Homo sapiens* targets, and database-specific confidence thresholds were applied to filter predicted targets. For TargetNet, targets with a predicted probability ≥ 0.5 were retained as higher-confidence predictions, consistent with thresholds commonly used in previous target-prediction studies [35]. For TCMSP, all available *Homo sapiens* targets associated with each candidate compound were retained without applying an additional numerical cutoff [36]. For HERB, human ingredient–target associations supported by curated reference or experimental evidence were retained [37]. For CTD, only chemical–target associations supported by at least two curated PubMed references were retained to increase the level of literature support [38]. Targets obtained from the four databases were subsequently standardized to UniProt gene identifiers, merged, and deduplicated to generate a unified target set. Finally, 3083 compound-associated targets were obtained from the 11 candidate compounds.

The Gene Cards database, DisGeNET database, and CTD were used to search for analgesic and anti-inflammatory targets. The keywords “pain” or “inflammation” were used to search for potential targets of analgesia and anti-inflammation, and duplicates were deleted after combining the search results, which were targets related to analgesia and anti-inflammation. Due to the large number of targets retrieved from the GeneCards database, the top 1000 targets ranked by relevance score were selected for subsequent analysis. Targets from DisGeNET and CTD were retrieved without additional filtering and merged with the GeneCards results after removing duplicates, yielding the disease-related target set.

**Figure 4:**
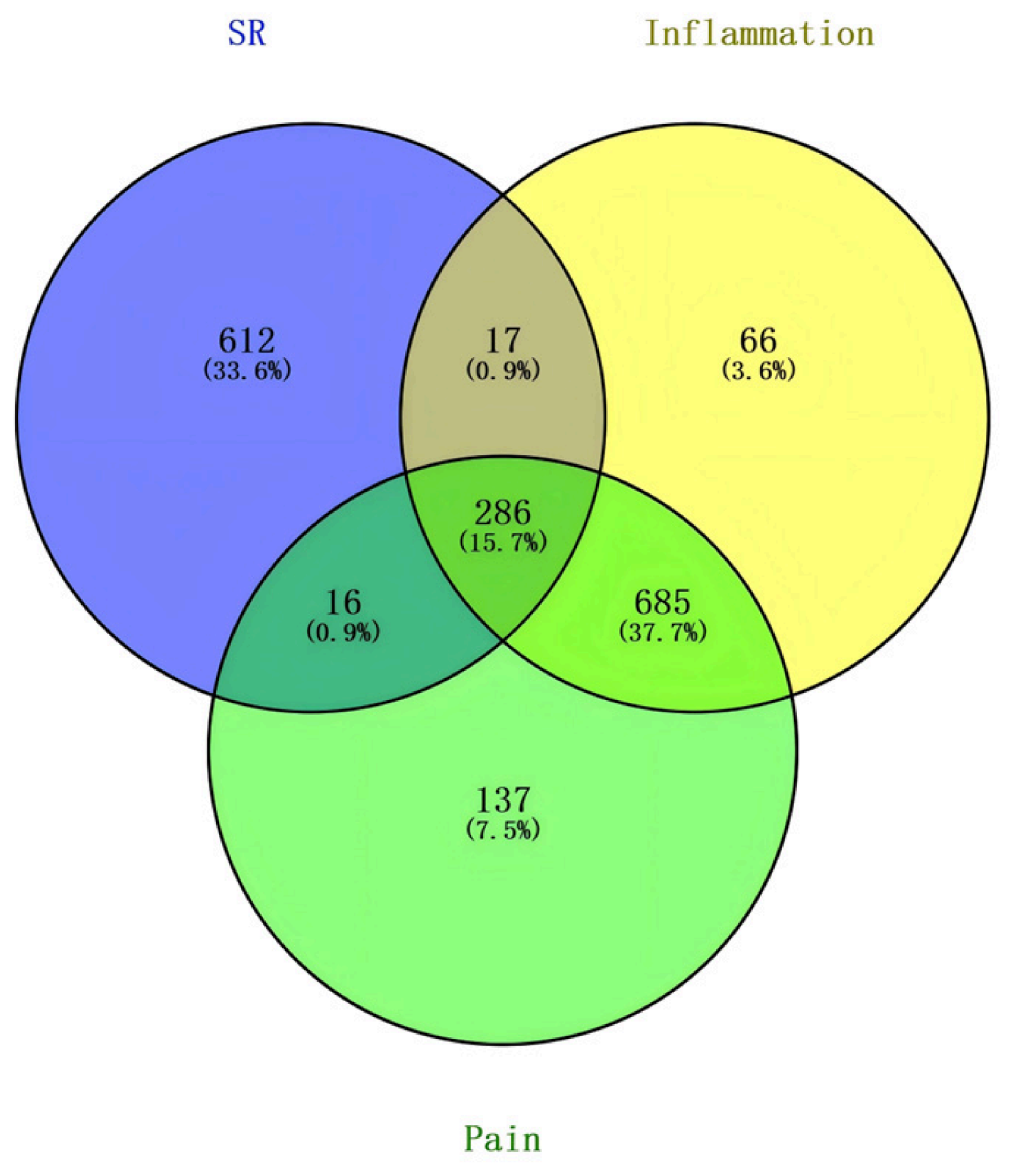
Venn plot of the intersecting targets.

The targets of the active compounds in SR and the targets of analgesic and anti-inflammatory agents were taken to be intersected. Meanwhile, the intersecting targets were obtained by working the Venn plot (Figure 4) using the Venny 2.1 platforms. Finally, 286 common targets were identified as candidate targets.

#### 3.2.2. PPI Network Construction and Analysis

**Figure 5:**
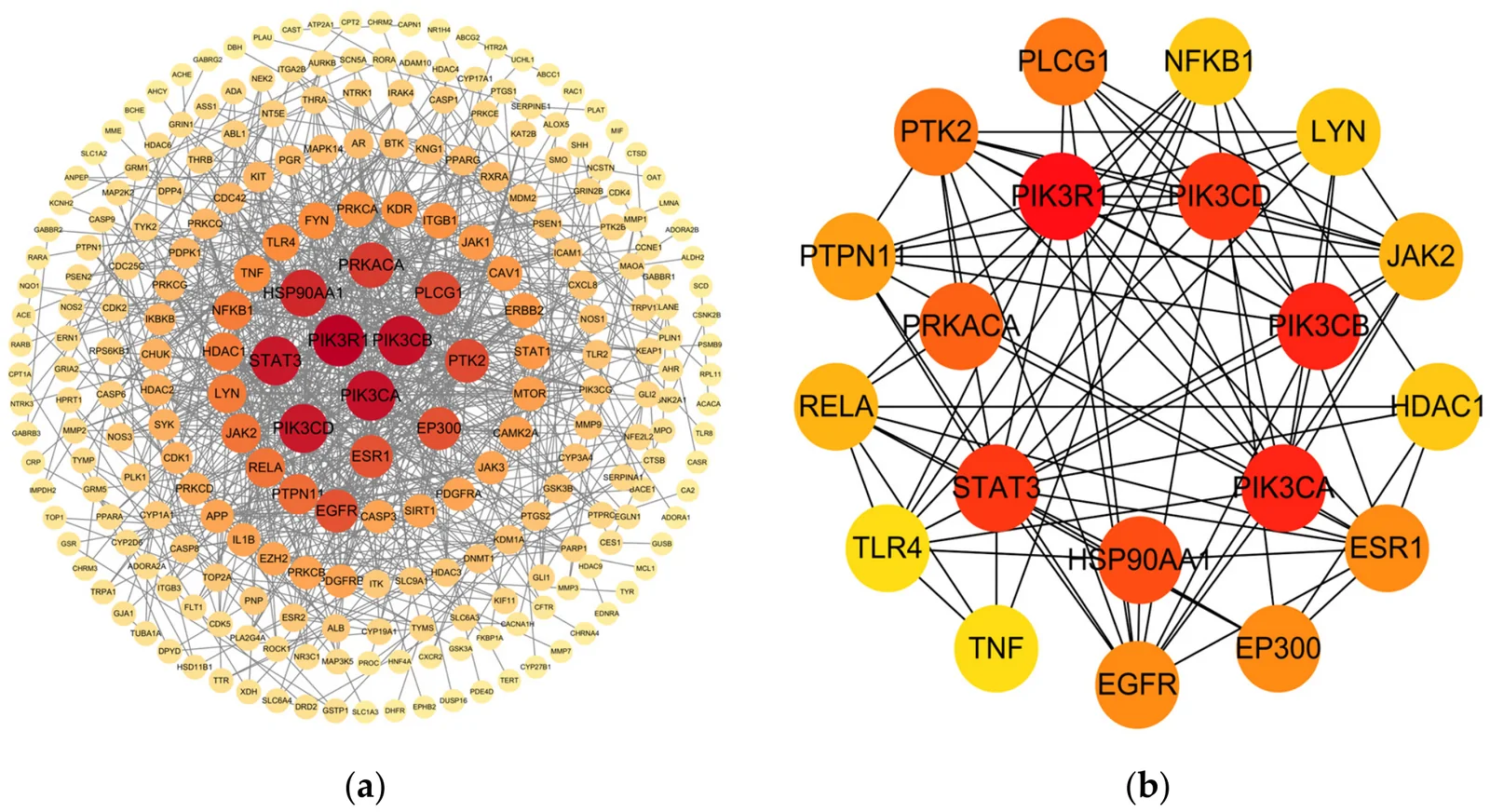
PPI network and core target analysis. (**a**) PPI network of the candidate common targets; node size and color intensity represent the Degree Centrality (DC) values, with larger and darker nodes indicating higher DC values. (**b**) The top 20 core targets ranked according to DC values.

The 286 targets were imported into the STRING database, with Homo sapiens selected as the species and the interaction score set to the highest confidence level (>0.900). Unconnected nodes were hidden, while all other parameters were kept at their default settings [39]. The analysis results of PPI were imported into Cytoscape 3.10.1 software to obtain the PPI network (Figure 5a). Parametric analysis was performed using the Analyze Network plug-in software, which calculates the values of Betweenness Centrality, Closeness Centrality, and Degree Centrality (DC), and the nodes are sorted in order of size and color depth in the graph. Node size and color intensity were mapped to DC values for visualization. Degree Centrality reflects the number of direct interactions (edges) connecting a given node to other nodes in the network, which was used as the primary criterion for ranking and selecting core targets. This approach is widely adopted in network pharmacology studies, as nodes with high DC values are generally considered hub nodes that may play critical roles in the biological network [40]. Then, we pick the top 20 based on DC values; namely, *PIK3R1*, *PIK3CA*, *PIK3CB*, *STAT3*, *PIK3CD*, *HSP90AA1*, *PRKACA*, *PLCG1*, *PTK2*, *ESR1*, *EP300*, *EGFR*, *PTPN11*, *JAK2*, *RELA*, *HDAC1*, *LYN*, *NFKB1*, *FYN*, and *TLR4* were used as core targets (Figure 5b).

#### 3.2.3. GO Enrichment Analysis and KEGG Pathway Analysis of Key Targets

**Figure 6:**
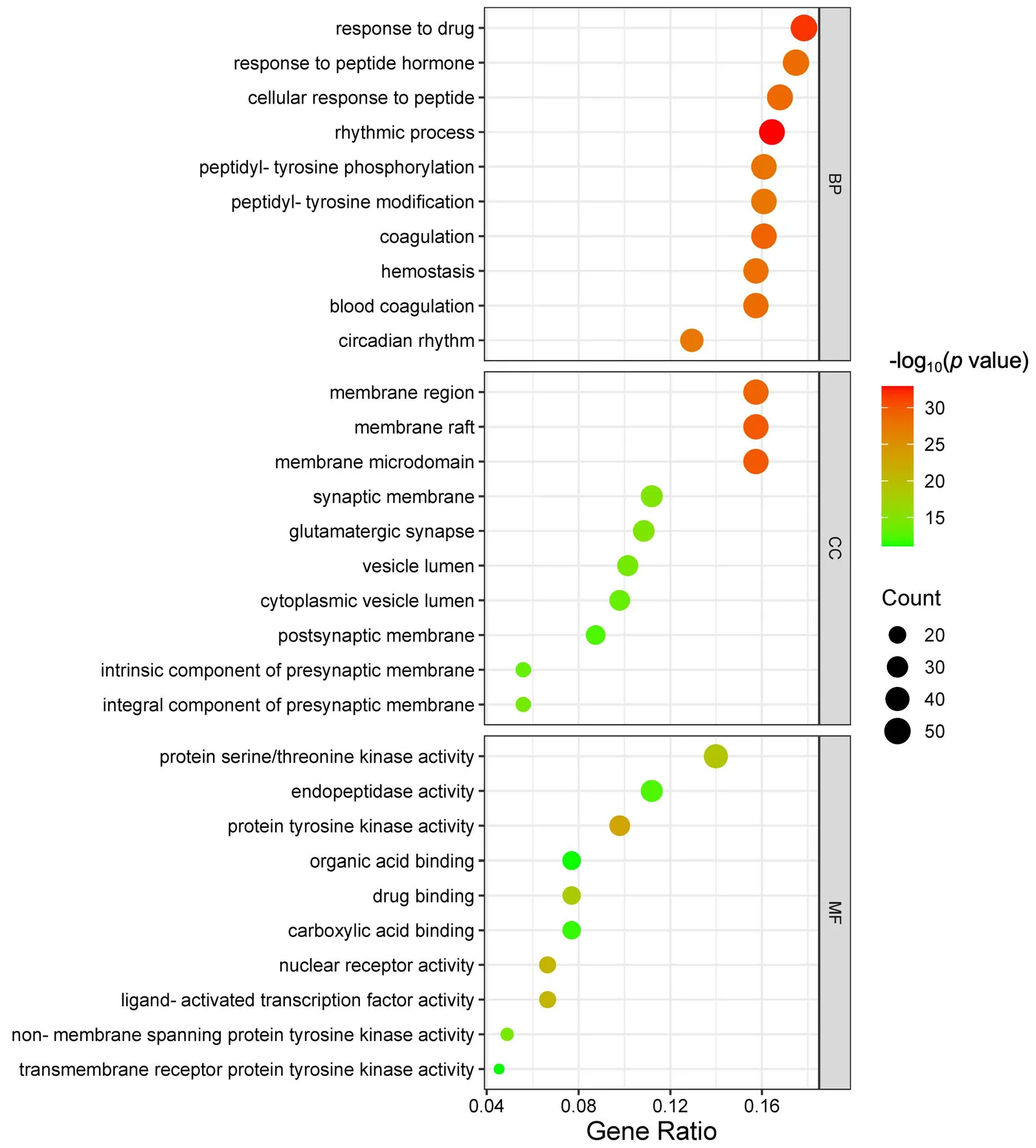
Biological processes enriched in the genes targeted by the 20 active components (the size and color of nodes represent the number of genes and *p* values of biological processes).

GO enrichment analysis was proceeded on the 20 core targets obtained utilizing the DAVID database [41]. GO enrichment results were analyzed to be mainly enriched for biological processes (BPs) related to response to drugs, response to peptide hormones, cellular response to peptides, rhythmic processes, peptidyl-tyrosine phosphorylation, peptidyl-tyrosine modification, coagulation, hemostasis, blood coagulation, circadian rhythm, etc. Cellular components (CCs) included membrane regions, membrane rafts, membrane microdomains, synaptic membranes, glutamatergic synapses, vesicle lumens, cytoplasmic vesicle lumens, postsynaptic membranes, intrinsic components of presynaptic membranes, synaptic intrinsic components of the presynaptic membrane, intrinsic components of the presynaptic membrane, etc. Molecular function (MF) entries mainly involved protein serine/threonine kinase activity, endopeptidase activity, protein tyrosine kinase activity, organic acid binding, drug binding, carboxylic acid binding, nuclear receptor activity, ligand-activated transcription factor activity, non-transmembrane protein tyrosine kinase activity, transmembrane receptor protein tyrosine kinase activity, etc. Figure 6 shows some of these important entries.

**Figure 7:**
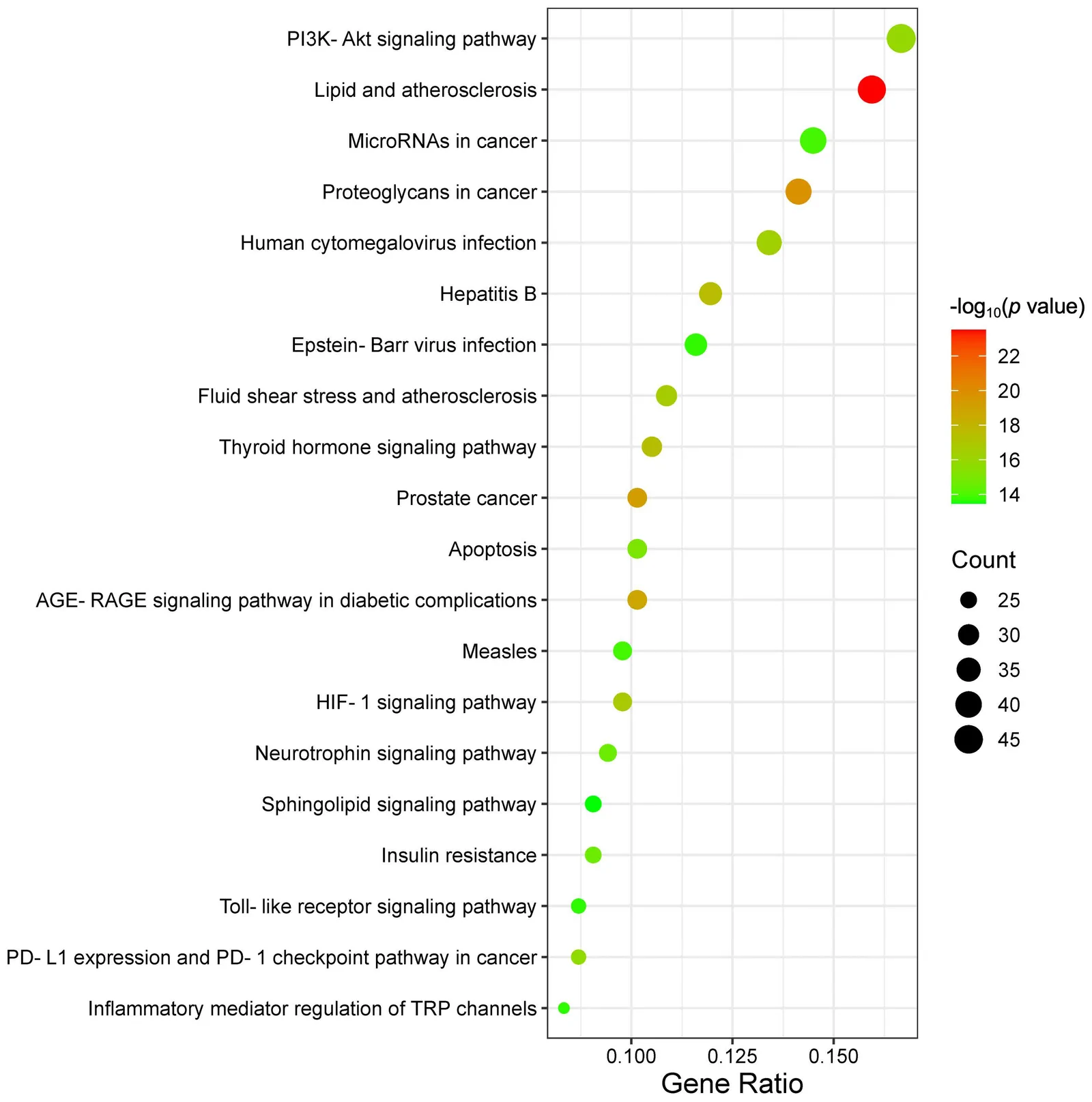
Bubble diagram of the top 20 pathways based on KEGG analysis (the size and color of nodes represent the number of genes and *p* values of biological processes).

The results of KEGG analysis showed that 197 KEGG signaling pathways are enriched by setting a *p* value < 0.01, and the top 20 pathways are displayed in Figure 7. These pathways mainly include the phosphoinositide 3-kinase–protein kinase B (PI3K-Akt) signaling pathway, lipids and atherosclerosis, microRNAs in cancer, proteoglycans in cancer, human cytomegalovirus infection, Hepatitis B, Epstein–Barr virus infection, fluid shear stress and atherosclerosis, the thyroid hormone signaling pathway, prostate cancer, apoptosis, the advanced glycation end products–receptor for advanced glycation end products (AGE-RAGE) signaling pathway in diabetic complications, measles, the hypoxia-inducible factor 1 (HIF-1) signaling pathway, the neurotrophin signaling pathway, the sphingolipid signaling pathway, insulin resistance, the toll-like receptor signaling pathway, the programmed death-ligand 1 expression and programmed death-1 checkpoint (PD-L1 expression and PD-1 checkpoint) pathway in cancer, regulation of TRP channels by inflammatory mediators, etc. These results hint that the active ingredients of SR may exert therapeutic effects by targeting these signaling pathways.

#### 3.2.4. Compound–Target–Pathway Network Construction

**Figure 8:**
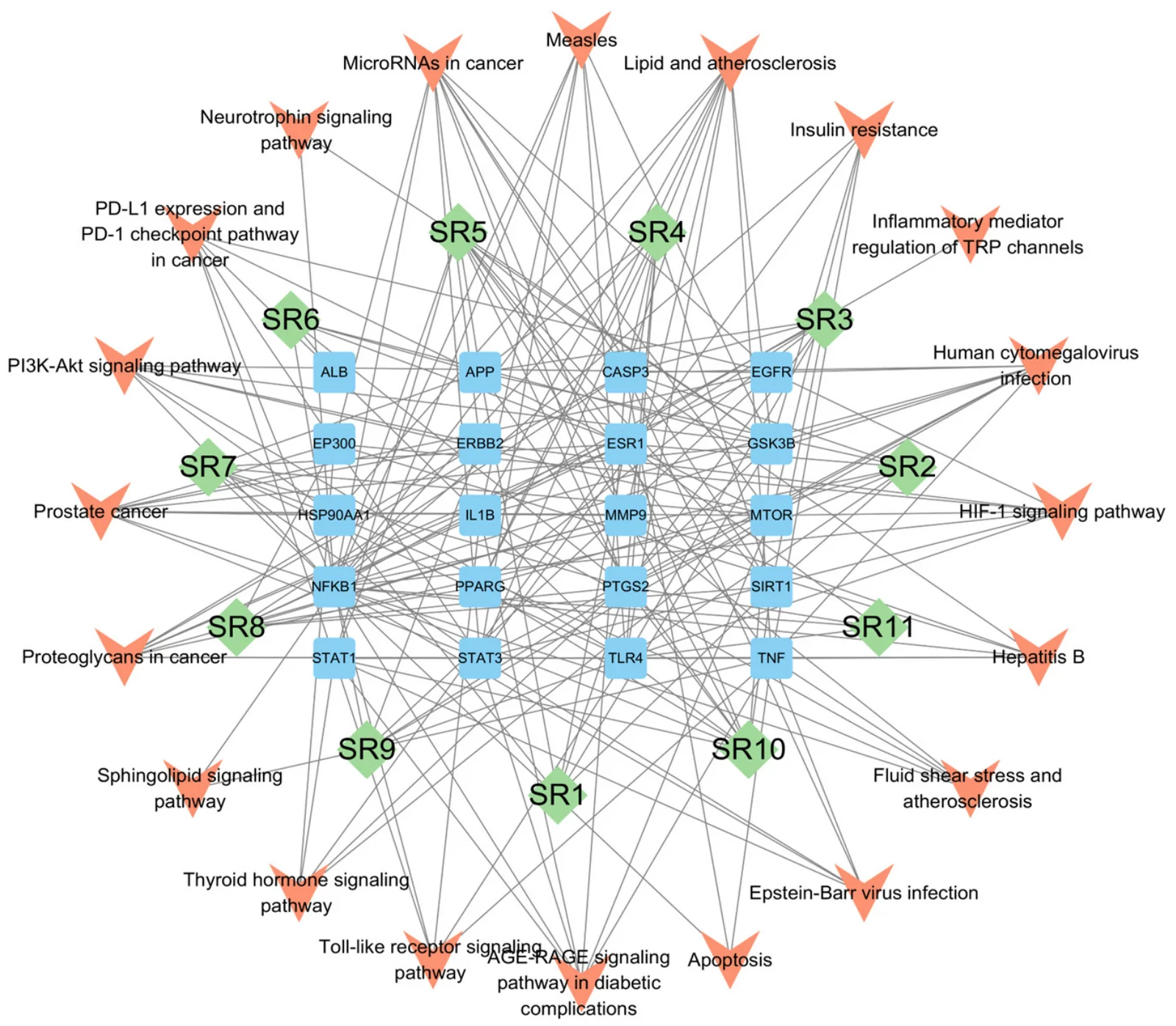
The “compound–target–pathway” network included 11 active components (in green rhomb), 20 targets (in bule quadrilaterals), and 20 enriched pathways (in orange arrows).

The important compounds, key targets, and pathways derived above were used to construct a “compound–target–pathway” network (Figure 8) using Cytoscape 3.10.1 software, and the degree values of compounds, target proteins, and signaling pathways were used as references [42]. Compounds were ranked by Degree Centrality (top three selected), targets were identified via a two-step PPI-based filter (degree > 10 and involvement in ≥2 pathways), and pathways were selected using KEGG significance (adjusted *p*-value, top three). The relatively high connectivity suggested that these compounds may be active substances of SR that exert pharmacological effects. The results suggest that *TNF*, *IL1B*, *ALB*, *EGFR*, *CASP3*, *STAT3*, *HSP90AA1*, *ESR1* and *NFKB1* may play important roles in anti-inflammatory and analgesic effects. Trans-3-Nonen-2-one, benzaldehyde, and Octanoic acid may be the putative bioactive compounds in the anti-inflammatory and analgesic effects of SR. Furthermore, this therapeutic mechanism might be implicated in the PI3K-Akt signaling pathway, apoptosis, and the HIF-1 signaling pathway. The therapeutic efficacy of TCM is generally considered to arise from the synergistic interactions among multiple compounds, targets, and signaling pathways [43,44]. The “compounds–targets–pathway” network [45] was constructed to clarify that different targets of different compounds in SR play different roles in different signaling pathways.

### 3.3. Molecular Docking Analysis

**Figure 9:**
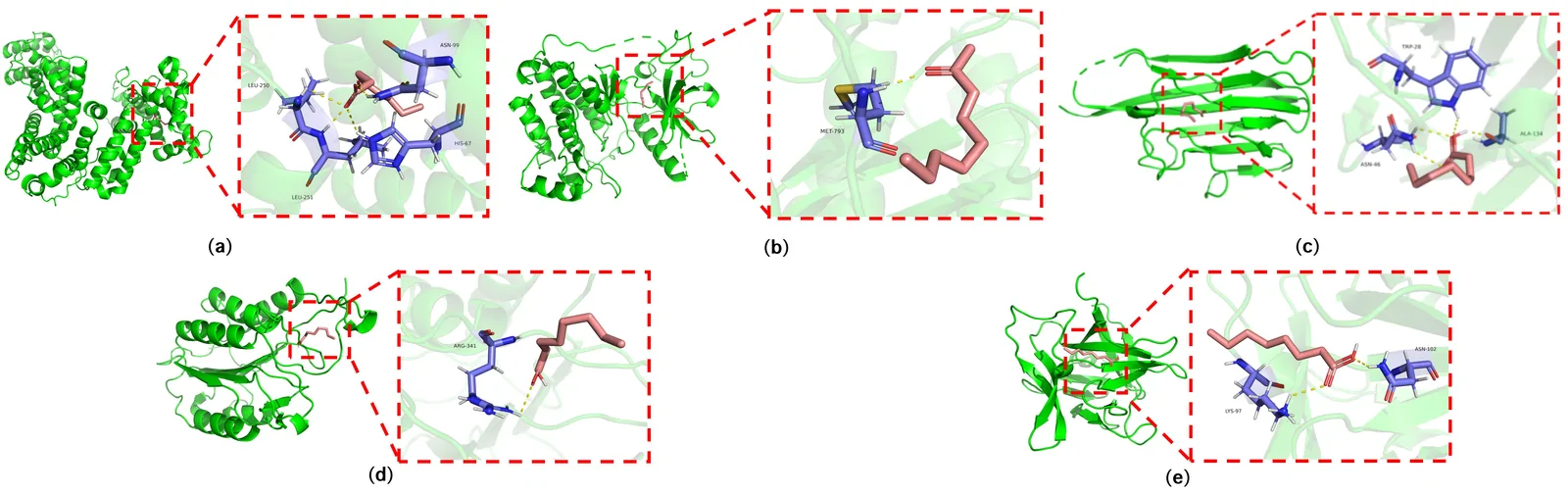
Molecular docking results of the representative docking complex of key targets and compounds: (**a**) ALB–Octanoic acid; (**b**) EGFR–trans-3-Nonen-2-one; (**c**) TNF–Octanoic acid; (**d**) CASP3–Octanoic acid; (**e**) IL1B–Octanoic acid.

The top five core targets (*TNF*, *IL1B ALB*, *EGFR*, *CASP3*) were selected for molecular docking validation with major differential compounds. The binding energies of the SR main active ingredients and the core targets were all negative, with good binding affinity, and the smaller the value, the greater the binding affinity [46], as shown in Table S5. The interaction pattern analysis of the lowest-binding-energy combinations from PyMOL revealed that the main interaction types were hydrogen bonding forces (Figure 9). For example, Octanoic acid was able to generate hydrogen bonding force with amino acids 99 ASN, 250 LEU, 251 LEU, and 67 HIS on ALB; Octanoic acid was able to generate hydrogen bonding force with amino acid 341 ARG on CASP3; trans-3-Nonen-2-one-2 was able to generate hydrogen bonding force with amino acid EGFR on MET amino acid No. 793; Octanoic acid generates hydrogen bonding force with LYS No. 97 and ASN No. 102 on IL1B; Octanoic acid generates hydrogen bonding force with ASN No. 46, ALA No. 134 and TRP No. 28 on TNF. Figure 8 displays the representative molecular docking results of the most stable pair of each key target and corresponding component.

## 4. Discussion

Previous studies have demonstrated that the chemical composition and pharmacological properties of SR can vary according to growth pattern and geographical origin. Fuchino et al. reported compositional differences among cultivated, wild, and commercial SR using UHPLC/MS, whereas Xu et al. further showed that different growth patterns were associated with differences in both chemical composition and pharmacological activity, with volatile oils identified as important contributors to the observed variation [4,8]. Consistent with these findings, the present study revealed clear differences in the volatile chemical profiles of SR associated with growth pattern, cultivation duration, and geographical origin. Importantly, our results extend previous studies by comparatively evaluating these three quality-associated factors within a single HS-GC-MS/MS-based analytical framework.

The observed differences in volatile profiles may reflect variations in plant developmental status, cultivation practices, and environmental adaptation. Changes in cultivation duration may influence secondary metabolism during plant growth and development, whereas geographical variation may be associated with environmental factors such as temperature, light, soil conditions, and other ecological influences. Recent studies have likewise emphasized the need for multidimensional quality evaluation of SR, since variation in chemical constituents may affect its biological activity and overall quality [8,47]. Notably, several compounds were repeatedly identified across different comparison groups in the present study, suggesting that they may be sensitive to multiple quality-related factors and could therefore represent candidate quality-associated volatile compounds.

The network pharmacology and molecular docking analyses further suggested that trans-3-Nonen-2-one, benzaldehyde, and Octanoic acid may be associated with inflammation- and pain-related targets and pathways. These findings are consistent with recent studies indicating that the pharmacological activity of SR may involve multiple chemical constituents acting through multiple targets and pathways. However, the predicted compound–target relationships in the present study should be regarded as hypothesis-generating and require further experimental validation.

From a quality control perspective, recent studies have increasingly explored multi-component and biological–chemical integrative strategies for the comprehensive evaluation of SR [47]. The present findings provide complementary information by incorporating volatile chemical variation into the evaluation framework. Nevertheless, the identified volatile compounds should currently be considered candidate quality-associated markers. Their quantitative reproducibility, chemical stability during processing and storage, and relationships with pharmacological efficacy require further validation before they can be considered for routine quality control or future pharmacopoeial applications.

## 5. Conclusions

In this study, HS-GC-MS/MS combined with multivariate statistical analysis was applied to characterize the volatile chemical profiles of SR with different growth patterns, cultivation durations, and geographical origins. Seven differential volatile compounds were identified as potential markers for distinguishing wild and cultivated SR, while 13 and 11 compounds were associated with differences in cultivation duration and geographical origin, respectively. By integrating the differential compounds identified across the three comparisons, 11 recurrent candidate compounds were selected for subsequent network pharmacology and molecular docking analyses. The results suggested that trans-3-Nonen-2-one, benzaldehyde, and Octanoic acid were predicted to interact with potential key targets such as *TNF*, *IL1B*, *ALB*, *EGFR*, and *CASP3.* These targets were significantly enriched in pathways including the PI3K-Akt signaling pathway, apoptosis, and the HIF-1 signaling pathway, suggesting that these compounds may be associated with pathways potentially relevant to the anti-inflammatory and analgesic activities of SR.

From a biological perspective, the observed variation in volatile profiles may reflect differences in plant growth, developmental status, cultivation practices, and environmental conditions. Cultivation duration may affect the accumulation of volatile metabolites through changes in plant development and secondary metabolism, whereas geographical variation may be associated with differences in environmental conditions and ecological adaptation. Notably, several compounds were repeatedly identified across different comparison groups, indicating that they may be sensitive to multiple quality-related factors and may therefore represent potential quality-associated volatile markers. However, the mechanisms underlying the differential accumulation of these volatile compounds remain to be clarified through targeted metabolomic, physiological, and biosynthetic studies.

From a quality control perspective, the present study provides complementary chemical information beyond the current pharmacopoeial marker-based evaluation of SR, which primarily focuses on a limited number of non-volatile constituents. The characterization of volatile chemical variation associated with growth pattern, cultivation duration, and geographical origin provides an additional dimension for understanding the quality heterogeneity of SR. Nevertheless, the volatile compounds identified in this study should currently be regarded as candidate quality-associated markers rather than established quality control markers. Furthermore, independent validation using additional sample batches and quantitative analytical methods is required to assess their analytical reproducibility, chemical stability during processing and storage, quantitative consistency across batches, and relationships with pharmacological efficacy.

Overall, the integration of volatile chemical profiling with chemometric analysis, network pharmacology, and molecular docking provides a complementary strategy for the comprehensive evaluation of SR quality. Such an approach may contribute to the development of a more comprehensive and multidimensional quality evaluation system and provide supporting evidence for the future refinement of pharmacopoeial quality control of SR and other medicinal Apiaceae species.

## Data Availability

The data presented in this study are available on request from the corresponding authors.

## Supplementary Materials

The following supporting information can be downloaded at: https://www.mdpi.com/article/10.3390/metabo16090689/s1, Figure S1: Venn diagram of overlapping differential volatile compounds across comparisons of wild vs. cultivated SR, different cultivation durations, and geographical origins; Figure S2: PPI network diagram of target proteins; Table S1: The detailed information of SR samples; Table S2: Results of identification of volatile components of SR; Table S3: The list of the volatile compounds subjected to network pharmacology analysis; Table S4: Topological properties of target network; Table S5: Biological process of genes; Table S6: KEGG enrichment analysis of core targets; Table S7: The degree values of for compounds and targets in “Compound–Target–Pathway Network”. Table S8: Molecular docking binding energy of major active ingredients of SR to core targets (kcal/mol).

## Abbreviations

The following abbreviations are used in this manuscript:

ALB	Albumin
AGE-RAGE	Advanced glycation end products–receptor for advanced glycation end products
BP	Biological process
CASP3	Caspase-3
CC	Cellular component
EGFR	Epidermal Growth Factor Receptor
EI	Electron ionization
GC-MS	Gas chromatography–mass spectrometry
GO	Gene Ontology
HIF-1	Hypoxia-inducible factor 1
HPLC	High-performance liquid chromatography
HS-GC-MS	Headspace gas chromatography–mass spectrometry
IL1B	Interleukin-1 Beta
KEGG	Kyoto Encyclopedia of Genes and Genomes
LC-MS	Liquid chromatography–mass spectrometry
MF	Molecular function
OPLS-DA	Orthogonal partial least squares–discriminant analysis
PCA	Principal component analysis
PD-L1 expression and PD-1 checkpoint	Programmed death-ligand 1 expression and programmed death-1 checkpoint
PI3K-Akt	Phosphoinositide 3-kinase–protein kinase B
PLS-DA	Partial least squares–discriminant analysis
PPI	Protein–Protein Interaction
QC	Quality control
TCM	Traditional Chinese medicine
TNF	Tumor Necrosis Factor
SR	Saposhnikoviae radix
UHPLC/MS	Ultra-high-performance liquid chromatography/mass spectrometry
UHPLC-QTOF-MS	Ultra-high-performance liquid chromatography–quadrupole time-of-flight mass spectrometry
UPLC	Ultra-performance liquid chromatography
UPLC-MS/MS	Ultra-performance liquid chromatography-tandem mass spectrometry
VIP	Variable importance in projection
